# IoT and AI-Based Application for Automatic Interpretation of the Affective State of Children Diagnosed with Autism

**DOI:** 10.3390/s22072528

**Published:** 2022-03-25

**Authors:** Aura-Loredana Popescu, Nirvana Popescu, Ciprian Dobre, Elena-Simona Apostol, Decebal Popescu

**Affiliations:** Computer Science Department, University POLITEHNICA of Bucharest, RO-060042 Bucharest, Romania; nirvana.popescu@upb.ro (N.P.); ciprian.dobre@upb.ro (C.D.); elena.apostol@upb.ro (E.-S.A.); decebal.popescu@upb.ro (D.P.)

**Keywords:** neural networks, autism spectrum disorder, humanoid robots, drawing interpretation

## Abstract

In the context in which it was demonstrated that humanoid robots are efficient in helping children diagnosed with autism in exploring their affective state, this paper underlines and proves the efficiency of a previously developed machine learning-based mobile application called PandaSays, which was improved and integrated with an Alpha 1 Pro robot, and discusses performance evaluations using deep convolutional neural networks and residual neural networks. The model trained with MobileNet convolutional neural network had an accuracy of 56.25%, performing better than ResNet50 and VGG16. A strategy for commanding the Alpha 1 Pro robot without its native application was also established and a robot module was developed that includes the communication protocols with the application PandaSays. The output of the machine learning algorithm involved in PandaSays is sent to the humanoid robot to execute some actions as singing, dancing, and so on. Alpha 1 Pro has its own programming language—Blockly—and, in order to give the robot specific commands, Bluetooth programming is used, with the help of a Raspberry Pi. Therefore, the robot motions can be controlled based on the corresponding protocols. The tests have proved the robustness of the whole solution.

## 1. Introduction

Autism spectrum disorder is a condition of brain development that can appear before the age of 3 years [1]. Children diagnosed with autism have difficulties in expressing their feelings and communicating nonverbally. Through drawings, children can express emotions, and this is where the PandaSays mobile application is helpful for children diagnosed with autism; in order to improve the communication between parents or tutors and their children and to understand better their children’s emotional state, the application will offer them some indicators based on the machine learning algorithms. Currently, the model trained with MobileNet has an accuracy of 56.25%, and the database of drawings is constantly increasing.

The main feature of the application is the use of machine learning algorithms to predict the affective state of the child from his/her drawings. Analysis of the drawings could provide parents/tutors or therapists a profound understanding of the child’s psychological or mental state. In addition, drawings can aid the comprehension of the child’s personality. This is a gap that will be filled by the PandaSays application.

Other features contained by the application are label recognition for identifying objects, activities, products, and so on, an augmented reality module, a drawing module, a sign language module, and Google Text-To-Speech. The Sign language and Google Text-To-Speech modules might be helpful in the case of aphasia or different types of speech impairments.

The latest studies have demonstrated that humanoid robots are efficient in helping children diagnosed with autism. Recent studies have demonstrated that children are more motivated and interested in the interaction with a robot rather than with parents or a tutor. The robots can play an important role in children’s education, as articles [2,3,4,5] describe; teachers might use them in the classroom to plan lessons. The roles played by the robots encompass educational planning, teaching social skills to children, personalizing robots for every child, accompanying teachers in classroom, and using robots to increase the awareness of the child about how their behavior can influence other children.

The website “robokind.com” [6] is dedicated to using robots in children’s education; their employees use robots to develop social skills for children with autism. For example, they use the “Milo” robot to simulate facial expressions and teach core vocabulary. In addition, in the curriculum, there are included modules that are emotional, behavioral, and communication related. The modules have different levels of complexity, so, as the children make progress, they are transferred to a higher module. Murray Bridge High School has been using NAO humanoid robots (programmable robots developed by Softbank Robotics, Tokyo, Japan) in the classroom for over two years, accompanying children to different classes such as IT (information technology), social skills improvement, and yoga [7]. Long-term studies suggests that social robots are effective in helping children diagnosed with autism. KASPAR (Kinesics and Synchronization in Personal Assistant Robotics) and NAO robots helped children to develop and improve their social skills and communication. KASPAR has been created to represent an expressive robot and its way of communication is more predictable and repetitive, which makes engagement with the child easier. In addition, the KASPAR robot has helped children to start recognizing emotions and is being used in hospitals [8].

In article [9], the authors describe how they used the interaction of the NAO humanoid robot with children that were diagnosed with autism, by playing a game. The NAO robot shows the children some emotional poses developed with Choregraphe. An Android application was developed to control the robot via WIFI (Wireless Fidelity). In the application there is a button called “Manage Behaviours”, where the user can play the specific pose of the robot. There is also a “Refresh” button to reset the connection with the robot. The purpose of these behaviors is to teach the child how to interpret emotions and start learning expressing them. The emotions used for the NAO robot were happy, angry, scared, tired, disgusting, shy, sad, and loving. Sloboda, J. A. et al. explain how music can bring out emotions in the listener [10]. Other studies [11,12] have shown that several emotions, such as anger, happiness, fear, sadness, and anger, arise in those who listen to specific type of music or songs. In article [13], the authors explore the importance of music as a therapy to help children with autism. The aim of their research takes into consideration the number of times the therapist intercedes to help the child engage with the robot and the number of times the child imitates a robot’s dance movement.

Paper [14] illustrates how the use of the NAO robots improves the verbal and non-verbal communication of children with autism. In this research, four children from Welfare of Autistic Children (SWAC, Dhaka, Bangladesh) participated, and four sessions were held. The first session was referred to the introduction of the robot and the second session had the purpose of getting answers from the children. The NAO robot asked the children some of the following questions: “How old are you?”, “How are you?”, “What is your father’s name?”, “What is your name?”, and “What is your mother’s name?”. The third session was dedicated to physical activities, such as dancing and exercising, and the fourth session was about gathering feedback regarding session 2 and 3. After four weeks, the overall performance increased by 45% in the first child, 70% in the second child, 30% in the third child, and 75% in the fourth child.

The work in article [15] proposes to investigate the feedback received from the therapy session held with children diagnosed with autism by engaging with a robot that uses colors in different conditions. Four colors were used for gathering feedback: yellow, red, green, and blue. The child’s favorite color was kept as a goal for the task, and the one that was disliked by the child was eliminated. For the experiment, a Touch Ball was used that had incorporated a force sensor that measured three axial forces. The paper concluded that the feedback obtained from touching the device is more effective than the one resulting from the touching forces of the caregiver.

All of the papers mentioned above reflect the importance of humanoid robots in helping children with autism. The part that we are introducing is affective state interpretation from drawings, connecting it further to the humanoid robot, and using its capabilities to help children express themselves and communicate better with their parents and with other children. In addition, autism spectrum disorder can be diagnosed from early age (2 years). As soon as the child begins drawing, the “PandaSays” mobile application can help the parent/tutor to monitor the child’s affective state as early as possible.

In our previous work [16] we discussed the Android application PandaSays, which predicts the affective state of children with autism from their drawings, and with regard to integration with humanoid robots activating their movements. More specifically, in [16], the possibility of controlling the robot Alpha 1 Pro [17], from Ubtech company (Shenzhen, China), using Bluetooth communication protocol was mentioned, a subject that is explored in the current paper. The robot is used to perform some action based on the output received from the machine learning algorithm. For example, if the affective state that resulted from a drawing is “sad”, the robot will start dancing in order to distract the child from his current state and make them more joyful. In this context, the current paper reaches the following objectives: testing the PandaSays mobile application, discussing performance evaluation using deep convolutional neural networks and residual neural networks, establishing a strategy for commanding the Alpha 1 Pro robot without its native application and developing a robot module that includes communication protocols with the PandaSays application. The output of the machine learning algorithm involved in PandaSays is sent to the humanoid robot to execute some actions, such as singing, dancing, and so on. Alpha 1 Pro has its own programming language—Blockly—and in order to give the robot specific commands, Bluetooth programming is used in conjunction with a Raspberry Pi. Therefore, the robot motions can be controlled based on the corresponding protocols. After setting up the communication with the robot, the code for controlling the robot is written in Python 3. The other robots that will be used in PandaSays application are NAO (SoftBank) and Marty (robotical.io); these are used for testing the mobile application and to further discuss the performance evaluations.

The article has five sections. The first section is “Introduction”, followed by Section 2 in which the PandaSays mobile application is described, including performance tests by using deep convolutional neural networks and residual neural networks. Section 3 presents the Alpha 1 series Bluetooth communication protocol, emphasizing the whole strategy for establishing the Bluetooth serial communication between Raspberry Pi and Alpha 1P robot, using a SPP (Serial Port Profile) application. Section 4 shows a comparison between Raspberry Pi 4, Native Alpha 1P Android App, and BlueSPP Android App, underlying the evaluation results based on their connectivity time. The last section is dedicated to conclusions and future work.

## 2. Description of “PandaSays” Mobile Application and Performance Tests Presentation Using Deep Convolutional Neural Networks and Residual Neural Networks

PandaSays is a mobile application dedicated to children diagnosed with autism and for parents and tutors to help them better understand their children’s feelings and expressions [18]. It is an application where the child can draw. This is a simple action, but studies have shown that drawings can reveal emotions and feelings. Our app does more than that; we have tried to automate the understanding and interpretation of the emotion associated with the child by using a neural network. We train the network with a corpus of annotated drawings, and we present our results in the prediction of the obtained model for the affective state of the child.

**Figure 1:**
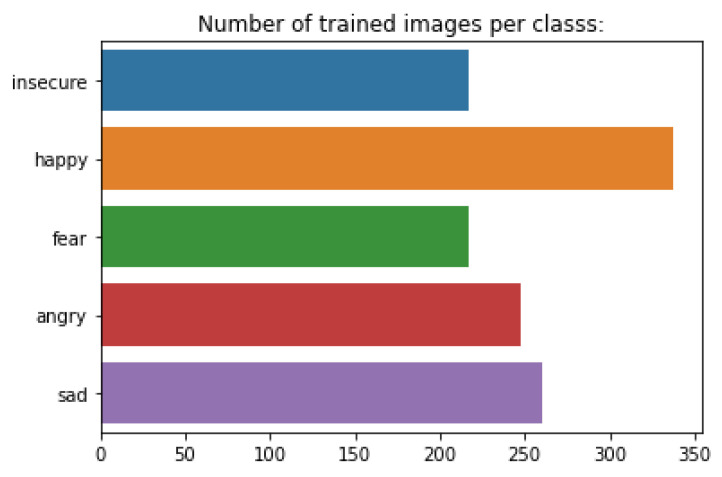
Number of trained images per class.

The dataset is composed of 1279 drawings, divided into 5 affective states, representing 5 classes: “happy”, “sad”, “fear”, “insecure”, and “angry” (Figure 1). The whole training data set has been labeled by a professional psychologist specialized in interpreting children’s drawings.

**Figure 2:**
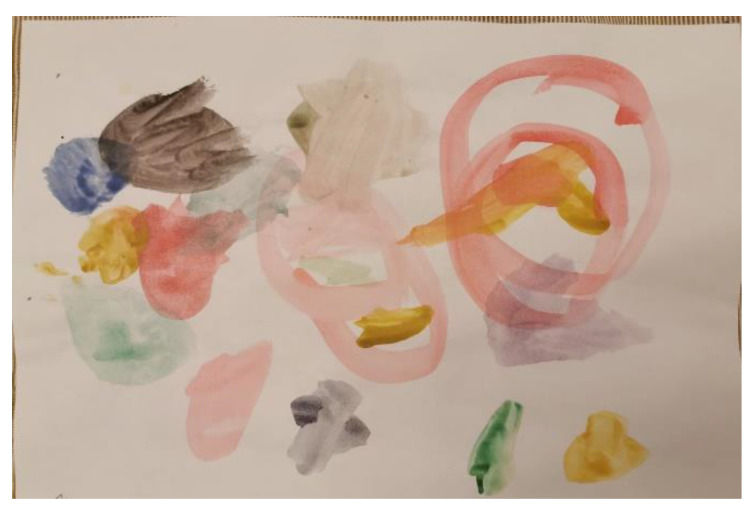
Drawing without the augmentation.

**Figure 3:**
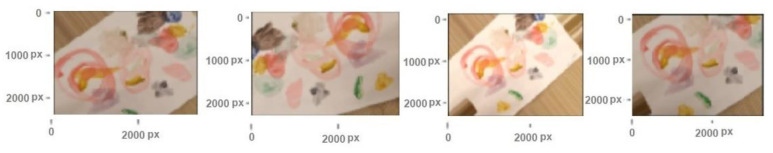
Drawing with Keras augmentation.

In our previous work [18], we realized a critical analyze of Convolutional Neural Networks and Feedforward Neural Networks, in order to find the best model to predict the affective state of the children from their drawings, and the conclusion was that MobileNet performed better than an artificial neural network. In the current work, increasing the number of drawings from the data set, we again trained the model using Convolutional Neural Networks and ResNet Neural Networks, and the new results will be also presented further in this article. The dataset was split as follows: 80% for training and 20% for testing. For the image data processing, Keras ImageDataGenerator [19] has been used. Figure 2 represents the original drawing and Figure 3 shows the drawings processed with ImageDataGenerator from the Keras library. The parameters and their values applied to the original image were the following:

- rotation_range = 40
- width_shift_range = 0.1
- height_shift_range = 0.1
- shear_range = 0.2
- zoom_range = 0.3
- horizontal_flip = True
- fill_mode = ‘nearest’
- brightness_range = [0.7, 1.2]

The rotation_range refers to the fact that the image is rotated randomly within the range of 40 degrees; width_shift_range means that the image is shifted horizontally (left or right) with 0.1 percent of the total image width, and height_shift_range, means that the drawing will be shifted vertically with 0.1 percent of the total image height. The image will be distorted along an axis with the range of 0.2 (shear_range), in order to simulate, to a computer, the way humans see things from different angles. The “zoom_range” parameter, refers to zooming inside the drawings. The drawings have horizontal_flip set to “True”, which means that the images will be randomly flipped horizontally. Parameter fill_mode is used for filling in the new pixels that were created, which can result after a rotation or a vertical/horizontal shift. The value “0.7” from the brightness_range indicates that the image will be darker, and the value 1.2 means that the image will be brightened.

First, for training the model, VGG16 (“Vision Geometry Group”) was used. For the training, the fully connected output layers of the model are not added and for weights, the “ImageNet” was utilized. The “ImageNet” dataset was considered because it contains approximatively 14 million images and every node is illustrated by hundreds and thousands of images [20]. This dataset is relevant, because it contains classes of animals, trees, and sports that children can draw. In addition, the database of drawings from PandaSays application has a small number of images, and transfer learning helped to reaching some valuable results. Further, three convolution dense layers with a ReLU (Rectified linear activation unit) activation function were added, in order to allow the model to learn more complex functions and to obtain better results.

For compiling the model, “Adam” (adaptive moment estimation) optimizer was used, which represents an optimization algorithm that operates sparse gradients on noise, with a learning rate of 0.001 and for loss, “categorical_crossentropy”. The VGG16 model summary is described in Table 1. Next, the first 20 layers of the network were set to be non-trainable and the result is presented in Table 2; there are 14, 714, and 688 non-trainable parameters and 2, 102, and 277 trainable parameters.

**Figure 4:**
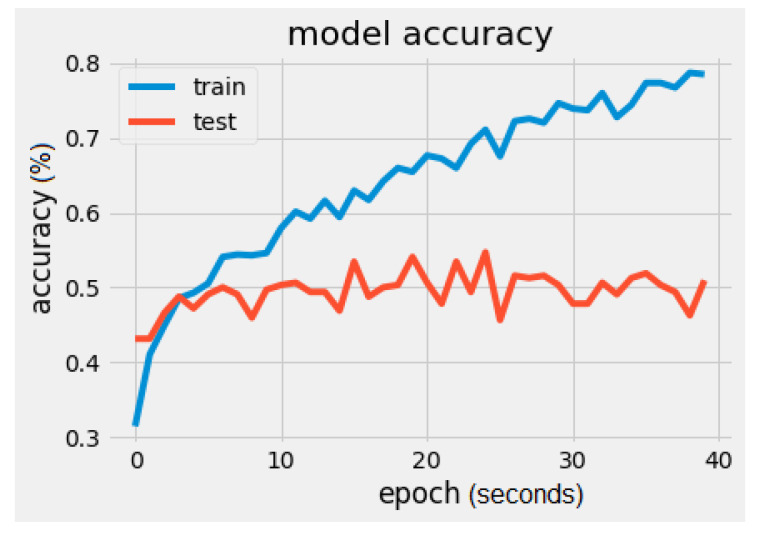
VGG16 model accuracy.

The results of the VGG16 model were loss = 1.8508 and accuracy = 50.9375% (Figure 4). The batch size was 16 and the number of epochs was 40.

**Figure 5:**
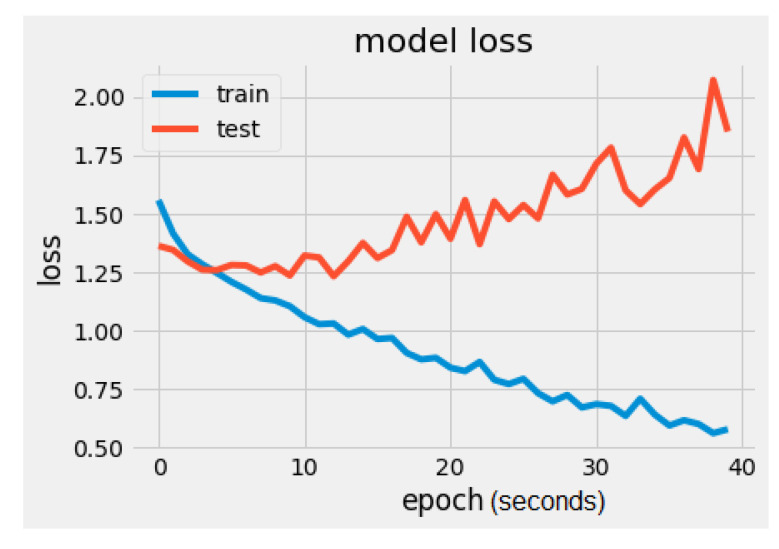
VGG16 model loss.

It can be noticed that the test loss (red line in Figure 5) is increasing while the training loss is decreasing. This indicates that the model is overfitting. The loss represents the difference between the expected output and the final output resulting from the machine learning model and is a double data type.

**Figure 6:**
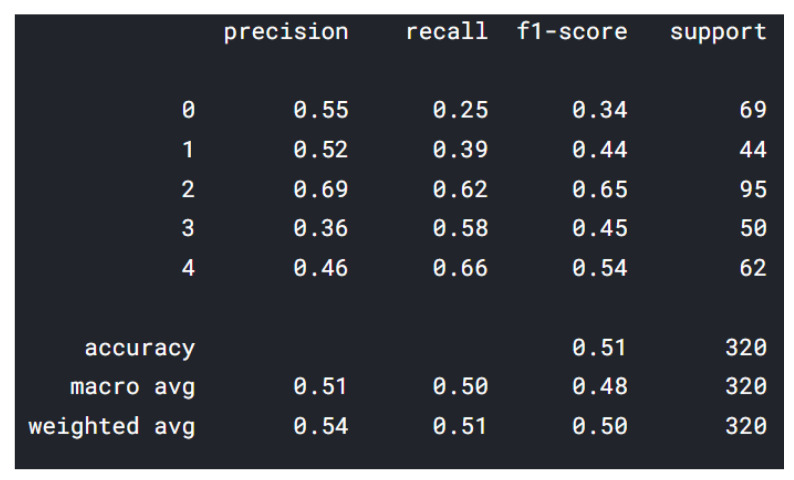
Vgg16 classification report.

In the classification report from Figure 6, the data shown from 0–4 represents 5 classes (applied on all classification reports—MobileNet and ResNet) described as follows: 0—insecure; 1—happy; 2—fear; 3—angry; 4—sad. The highest precision was represented by class “happy”, followed by “insecure”, and the lowest precision was represented by the class “angry”. “Happy” class has 337 drawings, while “angry” has only 248 drawings. One of the reasons for the lower precision “angry” is the color of images: some have multiple colors, and some are black/gray. We are using the RGB (red–green–blue) color space and for the future training we will take in consideration other color spaces, such as:

- CIE Lab (color space determined by the International Commission on Illumination)
- HSV (hue, saturation, value)
- HIS (hue, intensity, saturation)
- HSL (hue, saturation, kightness)
- YCbCr (green (Y), blue (Cb), red (Cr)) [21]

The best F1-score is obtained by the class “fear” and the lowest F1-score was represented by the class “insecure”, 0.34, which means that there are some false positives and some high false negatives. The best F1-scores are represented by the classes “fear” and “sad”. The F1 score has the following formula: F1 = 2 × (precision × recall)/(precision + recall) [22], and represents the mean of the precision and the recall, which is helpful to determine if there are large number of actual negatives.

For MobileNet, the “imagenet” dataset was used as weights; the batch size was 16 and the number of epochs was 40. The model contained the fully connected classifier and the summary is given in the Table 3.

**Figure 7:**
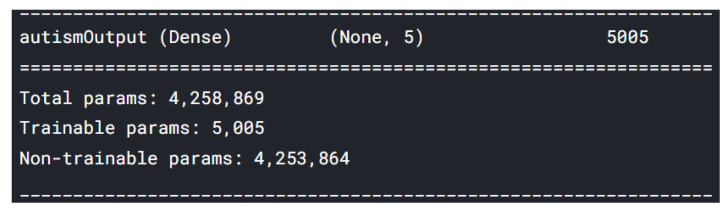
Last layer model summary.

Because we used a pre-trained model, we trained only the last layer and froze the other layers. The trainable parameters are from the last layer, named “autismOutput” (Figure 7).

**Figure 8:**
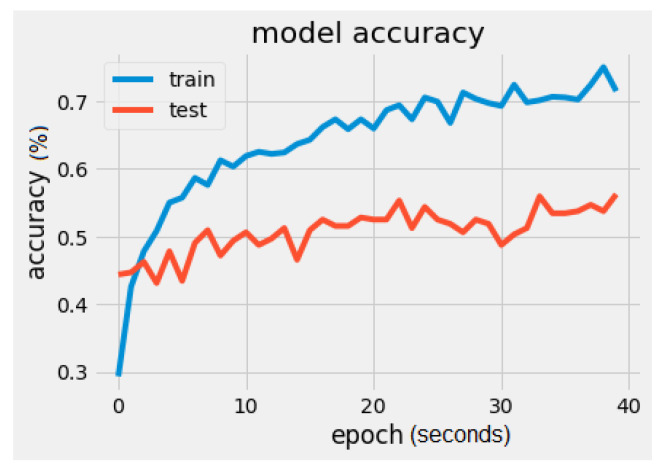
MobileNet model accuracy.

**Figure 9:**
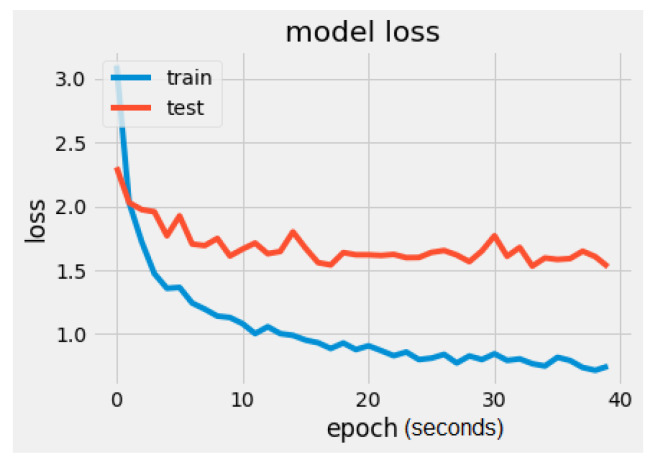
MobileNet model loss.

It can be noticed that there is a significant difference regarding the trainable parameters between the VGG16 model and the MobileNet model. MobileNet has 5005 trainable parameters and VGG16 has 2,102,277. The metrics values were loss = 1.5260 and accuracy = 56.2500%, as seen in Figure 8 and Figure 9. In Figure 9, the validation loss is a little greater than the training loss, which means that the model is performing well and is not overfitting. It is also important to mention that the dataset is constantly changing from the addition of new drawings.

**Figure 10:**
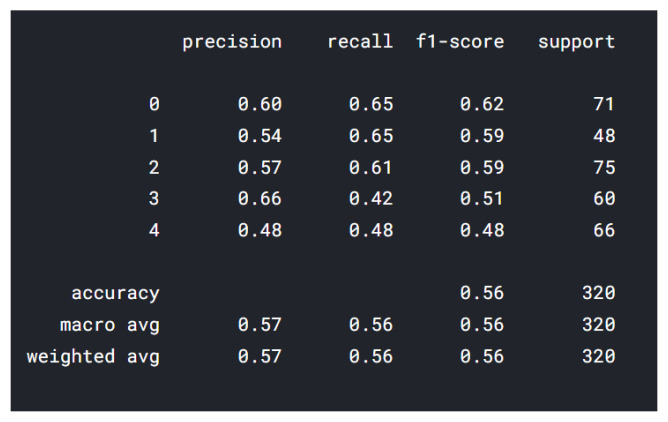
MobileNet classification report.

**Figure 11:**
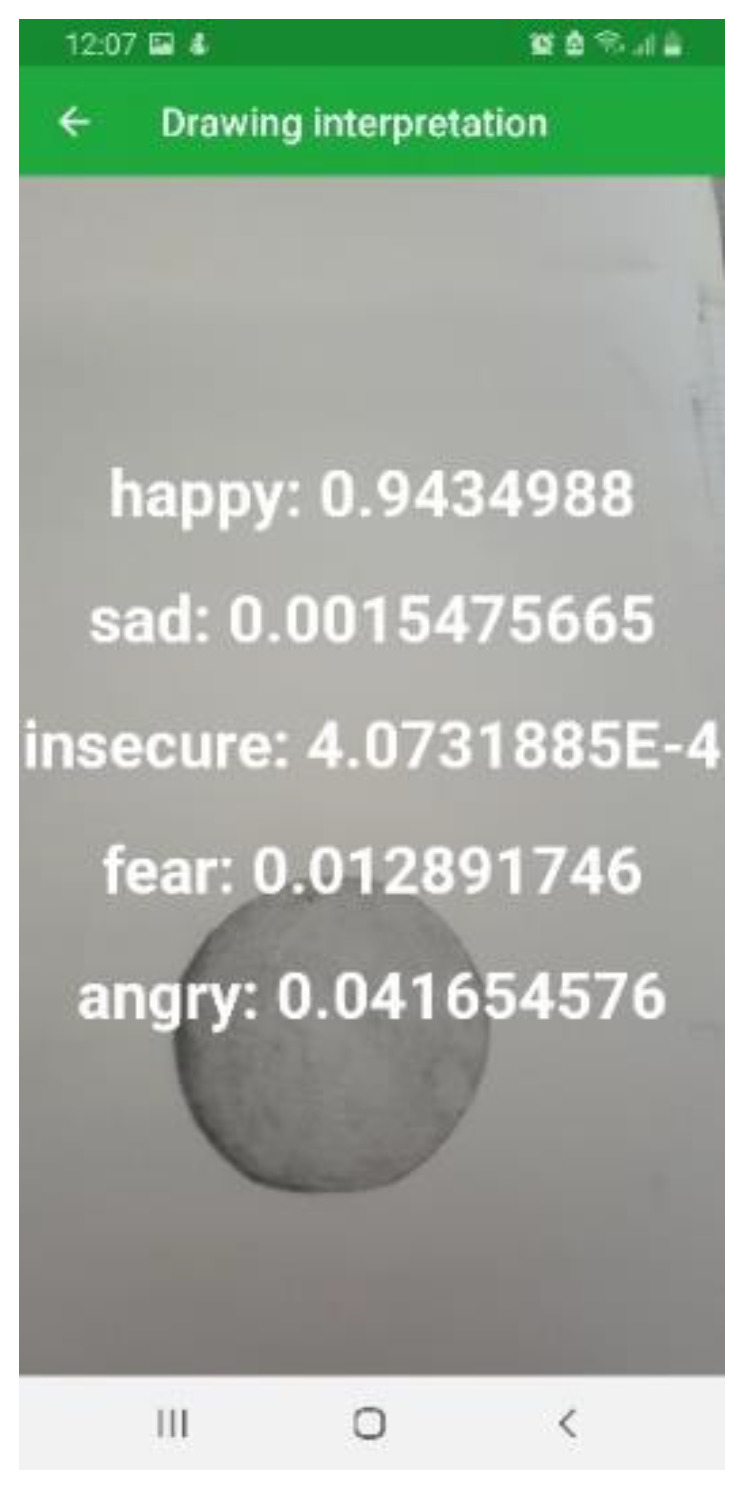
PandaSays drawing interpretation.

Figure 10 indicates that the highest precision was represented by the class “angry”—66%, followed by “insecure” with 60%, and the lowest precision was represented by the class “sad” at 48%. The highest F1-score is represented by the class “insecure” and the lowest by the class “sad”. As shown in Figure 11, the main output can be observed, which shows that the drawing represents a “happy” state, with 94% accuracy, when using PandaSays app with the MobileNet model.

Another neural network used for training the mode, was ResNet50 (Residual Neural Networks) from Keras. For weight, it was used the “imagenet” dataset, as input tensor it was used the input of shape (224,224,3), representing the width (224 pixels), height (224 pixels) and the number of channels (3), and a single pre-trained convolutional block was included (include_top = False). The ResNet base model is formed as follows: 

base_model = tf.keras.applications.ResNet50(weights = ‘imagenet’,include_top = False, input_tensor = input_tensor)

The first 143 layers of the network were set to non-trainable, as seen in the code bellow:

for layer in base_model.layers[:143]: layer.trainable = False

The pretrained model (base_model) will be connected with new layers of a new model. Global pooling, a flatten layer, and a dense layer was added, with a softmax classifier.

The model summary is presented in Table 4.

**Figure 12:**
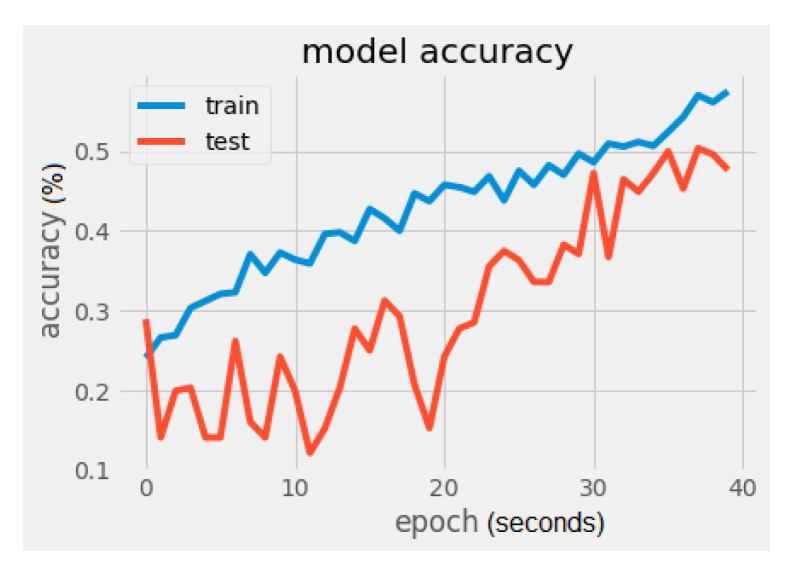
ResNet50 model accuracy.

**Figure 13:**
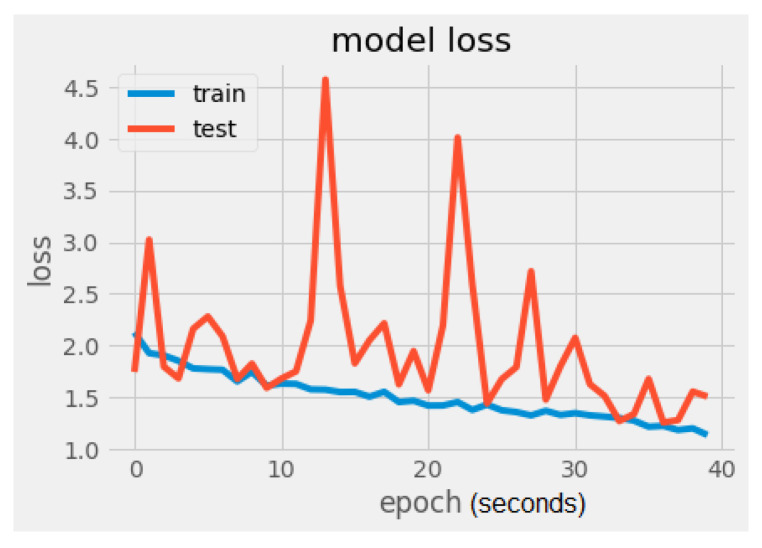
ResNet50 model loss.

The ResNet50 model’s loss was 1.5041 and the accuracy was 47.6562%. As an optimizer for compiling the model, RMSprop (Root Mean Square Propagation) was used from Keras, with a learning rate of: 2 × 10^−4^. The accuracy is displayed in Figure 12 and the model loss is presented in Figure 13. The validation loss is significantly greater than the training loss, which means that the model is overfitting.

**Figure 14:**
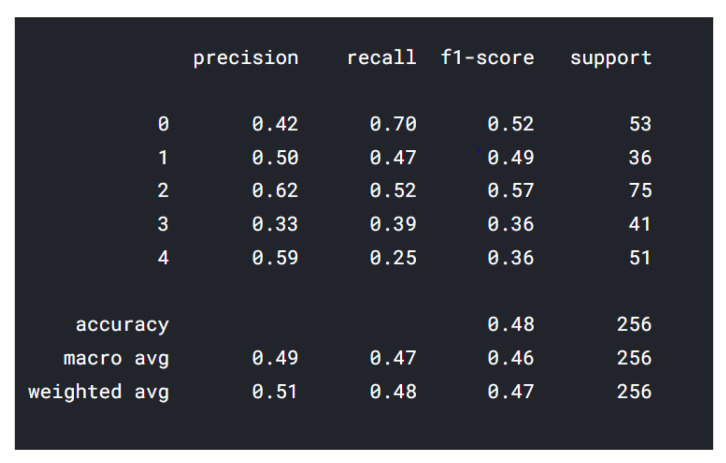
ResNet50 classification report.

Figure 14 illustrates that the highest precision was represented by the class “happy”—62%, followed by “sad” with 59%, and the lowest precision was represented by the class “angry”, with 33%. The highest F1-score is represented by the class “insecure” class and the lowest by the classes “sad” and “angry”—0.36.

As noticed in Table 5, the highest accuracy is obtained by MobileNet neural network with 56.25%, followed by VGG16 with 50.93%. Although the smallest accuracy is obtained by ResNet50 neural network, its loss is the smallest, followed by MobileNet’s loss of 1.5260%. For the PandaSays application, this was chosen as the model trained with MobileNet neural network because of its accuracy results.

In Table 6, it is shown that VGG16 is the most complex model, because it has the largest number of parameters (138 million) [23], followed by ResNet50 with over 23 million parameters [24] and MobileNet, which is the least complex model, with 13 million parameters [25]. In terms of latency, ResNet50 has the highest latency, and VGG16 has the lowest. ResNet50 has the lowest time of convergence and MobileNet has the highest time—422.074 seconds, regarding the time of convergence.

## 3. Alpha 1 Pro Server–Client Connection and Bluetooth Communication Protocol

One of the objectives of the current research is to present how the Alpha 1 Pro robot can be commanded without its native application and considering the integration with PandaSays application. Alpha 1 Pro has 16 servo joints, equivalent to humans’ joints. The robot can dance, move, fight. Based on interpretation of drawings, the robot will execute these actions, helping with children’s therapy and mood interpretation. In this section, the communication protocol will be presented based on the Bluetooth communication. The Alpha 1 Pro robot can be programmed through Bluetooth using the Bluetooth communication protocol [26].

The command format formula is:

**Header + Length + Command + [first parameter][second parameter][third parameter] + Check + End Character**

**BT handshake:**

Field Header (2B) is set to 0XFB 0XBF. Length (1B) represents the total number of bytes of (header + length + command + parameter + CHECK). Field Command (1B) refers to a specific command and Parameter (nB) represents one parameter at least. If the parameter is not needed, then the default value 0x00 is used. Field CHECK (1B) is composed of length + command + parameter and the End character (1B) is set to 0XED.

The following actions can be implemented by the robot, writing the specified code:

- Handshake (Read): FB BF 06 01 00 08 ED
- Obtaining an action list (Read): FB BF 06 02 00 08 ED
- Implementing an action list (Write): FB BF 06 03 00 09 ED
- Sound switch (Write): FB BF 06 06 00 08 ED
- Play control (Write): FB BF 06 07 00 08 ED-pause
- Heartbeat packet (Write): FB BF 06 08 00 08 ED
- Reading robot state (Write): FB BF 06 0A 00 10 ED
- Volume adjustment (Write): FB BF 06 0B 09 08 ED
- Powering off all servos (Write): FB BF 06 0C 00 12 ED
- Controlling all servo indicators (Write): FB BF 06 0D 00 08 ED
- Clock calibration (Write): FB BF 06 0E 08 ED
- Reading clock parameters (Read): FB BF 11 0F 06 18 ED
- Setting clock parameters (Write): FB BF 12 10 07 20 ED
- Reading the software version number of the robot (Read)
- Reading battery capacity of the robot (Read): FB BF 06 11 00 08 ED
- Controlling the motion of a single servo (Write): FB BF 08 23 03 13 ED
- Controlling the motion of multiple servos (Write): FB BF 08 23 03 12 ED
- Setting offset value of a single servo (Write): FB BF 08 26 03 12 ED
- Setting offset value of multiple servos (Write): FB BF 07 27 02 09 ED
- Play completion (Automatic report of BT): FB BF 05 31 00 05 ED
- Allowing change during play (Write/Automatic report of BT): FB BF 06 32 01 08 ED
- Completing action list sending (Automatic report of BT): FB BF 05 81 00 05 ED

These commands will be used further with the BlueSPP application to transmit to the robot one of these actions. More details about the BlueSPP communication with the robot is presented in Section 3.2.

Alpha 1P robot has dual-mode Bluetooth 3.0/4.0 BLE (Bluetooth Low Energy) + EDR (Enhanced Data Rate). Bluetooth Classic (or BR/EDR) is the Bluetooth radio that is mostly used in streaming applications and in the following devices/applications wireless headsets, wireless speakers, wireless printers and keyboards, and file transfers between devices. Bluetooth LE (low energy), is utilized in low-bandwidth applications that rarely need data transmission between devices. Bluetooth LE is known for its very low power consumption and is present in the following devices/applications: smartphones, computers and tablets [27], mobile payment applications, healthcare system, ticketing or access control applications.

Every Bluetooth chip is marked with a globally unique 48-bit address, which represents the Bluetooth address, device address or MAC (Media Access Control) address. This is identical in nature to the MAC addresses of Ethernet [27]. Some Bluetooth protocols are utilized in the same contexts as internet protocols. For connecting the raspberry pi device with Alpha 1P robot, it will be used RFCOMM (Radio frequency communication) protocol -set of transport protocols, providing emulated RS-232 (Recommended Standard 232) serial ports. RFCOMM provides mainly the same attributes as TCP (Transmission Control Protocol); the difference between the two is that TCP supports up to 65,535 open ports on a single machine, while RFCOMM only allows 30 [28].

### 3.1. Establishing Bluetooth Communication with Python

To provide access to Bluetooth system resources on GNU/Linux computers, we use PyBluez, which is a Python extension. The PyBlueZ module provides a high-level socket interface for establishing a connection between two Bluetooth devices. One acts like a client and the other as server. In our case, Raspberry pi is the server and Alpha 1P is the client. The Python code for establishing the client–server connection of the robot and Raspberry Pi can be found in the links provided in references [29,30].

### 3.2. Establishing Bluetooth Serial Communication between Raspberry Pi and Alpha 1P Robot Using a SPP Application

**Figure 15:**
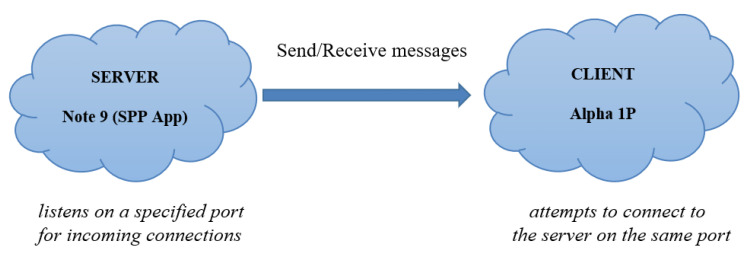
Server–client communication.

Bluetooth programming in Python is based on the socket programming model, a socket being an endpoint of a communication channel. When sockets are first created, they are not connected. In order to complete a connection, it must be called connect (client application) or accept (server application) [31]. For sending messages to Alpha 1P via Bluetooth, a Samsung Note 9 mobile phone and BlueSPP android application were used. The communication between the server and the client is illustrated in Figure 15.

**Figure 16:**
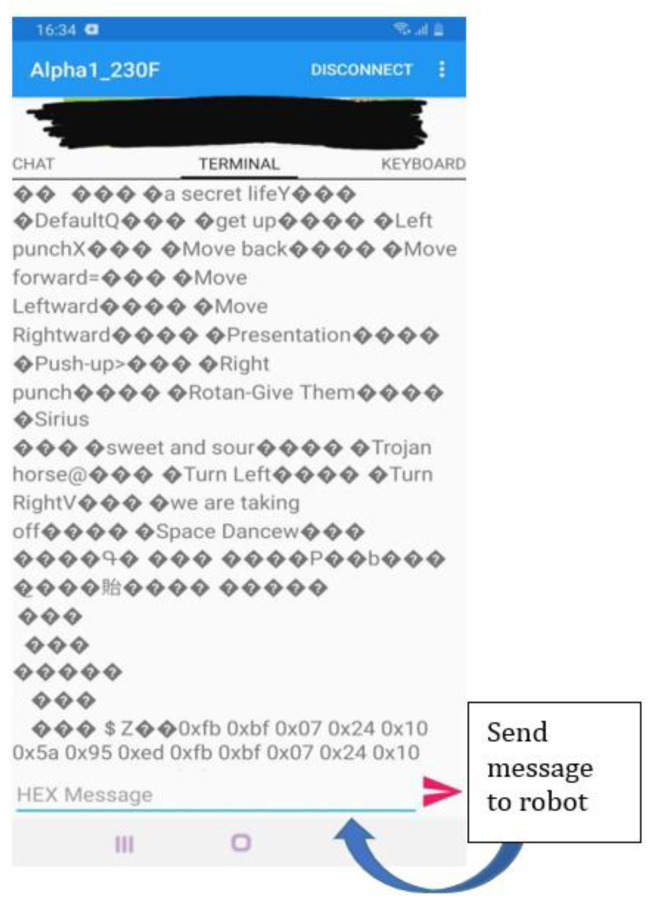
Message sending to robot through SPP android application.

According to the Bluetooth communication protocol document, the robot can perform numerous actions. For powering off of all the servos, we transmit to the robot the next command: FB BF 06 0C 00 12 ED (Figure 16).

In order to see the status of the robot, the next code is transmitted: FB BF 06 0A 00 10 ED. The send operation of the message is illustrated in Figure 16. The question marks in this figure are shown, because the numbers are sent randomly from Alpha1 Pro to the BlueSPP app, so the application is interpreting the bytes as ascii literals.

**Figure 17:**
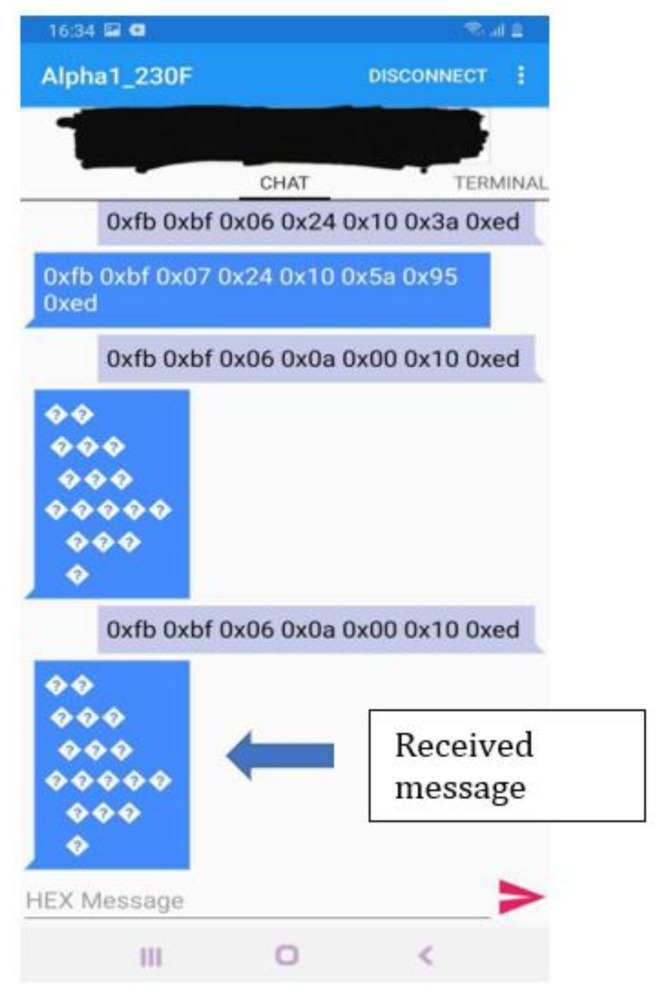
Response of the robot.

As it can be seen in Figure 17, the robot receives the next code associated to each status:

- FB BF 07 0A 00 01 12 ED (Speaker)
- FB BF 07 0A 01 00 12 ED (Play)
- FB BF 07 0A 02 80 93 ED (Volume)
- FB BF 07 0A 03 01 15 ED (Servo LEDs)

Figure 17 illustrates that the robot responded to the message sent using Bluetooth communication protocol.

## 4. Comparison of the Communication Times from Candidate Devices

**Figure 18:**
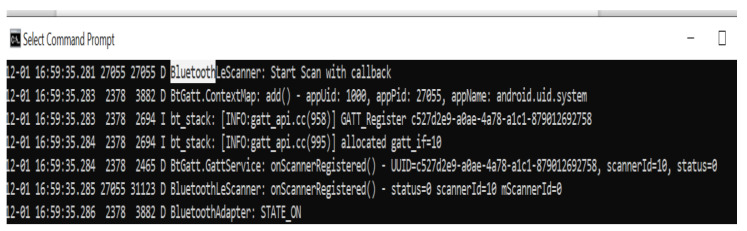
BlueSPP app execution time of action for Alpha 1P robot.

The BlueSPP android app is a Bluetooth Serial Port Profile communication tool. The device used was a Samsung S10+ Android phone. To measure the time, a digital watch was used. The required time to connect to the robot was 3.8 s. Bluetooth latency, representing Alpha 1P robot’s execution of a certain action, for BlueSPP Android app, on Samsung S10+, was 5 s (Figure 18).

**Figure 19:**
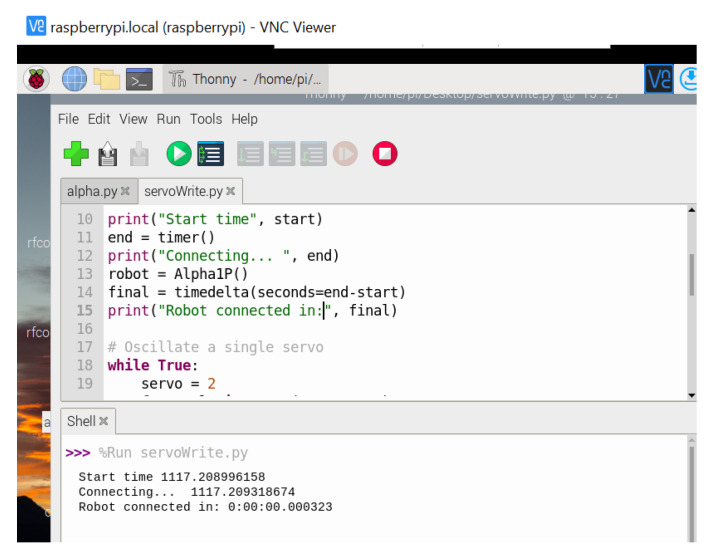
Raspberry Pi connection to Alpha 1P Robot.

For connecting the Raspberry Pi 4 to the Alpha 1P Robot, a Bluetooth adapter with frequency of 2.4–2.4835 (GHz) was used. The PyBluez library was used to establish a client-server connection. Figure 19 shows the time in seconds for the connection to the robot—3.23 s.

**Figure 20:**
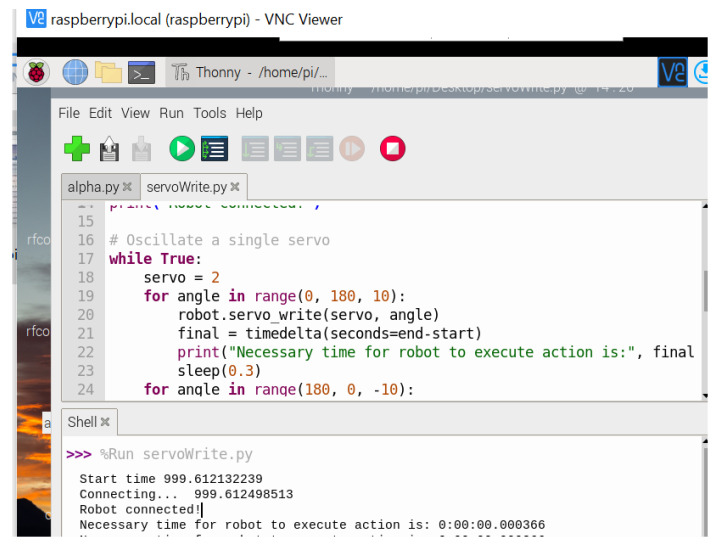
Execution time for Alpha 1P robot with Raspberry Pi.

The Bluetooth latency, representing Alpha 1P robot’s execution of a certain action, was 3.66 s for Raspberry Pi, as shown in Figure 20.

The Alpha 1P robot has its native android application called “Alpha 1” [32], which is available here: https://play.google.com/store/apps/details?id=com.ubt.alpha1s, accessed on 10 February 2022. The total time representing the successful execution of pairing and the connection to the robot was 28.18 s. The Bluetooth latency, representing Alpha 1P robot’s execution of a certain action, for the Android “Alpha 1” Android app on a Samsung S10+ was 31.22 s.

Table 7 presents the comparative results regarding the connectivity time of the robot. The best time is represented by Raspberry Pi (3.23 s) through the Bluetooth version, which is lower than the other devices.

Table 8 shows that the best time for the Alpha 1P robot to execute an action is obtained by Raspberry Pi, followed by BlueSPP Android App.

## 5. Conclusions

Humanoid robots play an important part in helping children diagnosed with autism. As several studies demonstrated, children are keener to interact with a robot than with their parents, doctors, or tutors. Robots can teach children how to express feelings, how to interact with other children, and how to communicate better. The interaction with the robot is one of the essential parts in the PandaSays application for the machine learning algorithm to be complete.

Raspberry Pi with PyBluez can be used to create a fast Bluetooth connection and to start programming a robot to execute some actions. The time difference represented on the Android devices is due to the Bluetooth latency as is well analyzed in [33].

The contributions brought by this paper are:

- In this paper, it was shown that the best trained model was the one trained with MobileNet, because of its highest accuracy—56.25%. MobileNet is the least complex neural network, with 13 million parameters, in comparison with VGG16 and ResNet50. Because of its low complexity and small size, MobileNet is more suitable for mobile applications. Those are the reasons why the MobileNet model was chosen to be used in PandaSays mobile application.
- Establishment of a control methodology for connecting the robot Alpha 1 Pro with PandaSays application, using Bluetooth communication protocol.
- Development of a robot module that includes the communication protocols with the app PandaSays, which will be used further to control the robot and send the machine learning output to it, in order to perform a specific action.
- Python module implementation for setting the client–server communication.
- The configuration setup of the Raspberry Pi and robot’s Bluetooth communication protocol, used to measure latency and connectivity time.
- The efficiency of using Raspberry Pi with PyBluez to create a client–server connection, represented by the lowest latency—3.66 s and by the connectivity time—3.23 s, which was faster than other devices (Android Device, BlueSPP app).
- Emphasis of the importance of the humanoid robots in helping children diagnosed with autism.

Given the small data set, we used a pretrained model and applied Transfer Learning. In future work, the next step will be to enlarge the data set and to apply the following algorithms to train the model: K nearest neighbor, decision tree, and support vector machines.

Another step for us would be to use the PandaSays application and one of the humanoid robots (Alpha, NAO, or Marty) in Autism Centers in Romania to gather feedback and improve the machine learning algorithm.

## Figures and Tables

**Table 1:** Vgg16 model summary.

Vgg16 Model Summary
Total params: 16,816,965
Trainable params: 16,816,965
Non-trainable params: 0

**Table 2:** Vgg16—model summary for first 20 layers.

Vgg16 Model Summary—First 20 Layers Non-Trainable
Total params: 16,816,965
Trainable params: 2,102,277
Non-trainable params: 14,714,688

**Table 3:** MobileNet model summary.

MobileNet Model Summary	
Total params	4,253,864
Trainable params	16,816,965
Non-trainable params	21,888

**Table 4:** ResNet50 model summary.

ResNet50 Model Summary	
Total params	49,722,757
Trainable params	49,468,037
Non-trainable params	254,720

**Table 5:** Neural network accuracy and loss comparison.

Neural Network	Testing Accuracy (%)	Training Accuracy (%)	Loss (Double Data Type)
MobileNet	56.25	70.56	1.5260
VGG16	50.93	73.93	1.8508
ResNet50	47.65	57.53	1.5041

**Table 6:** Neural network metrics comparison.

Neural Network	Complexity (Number of Parameters)	FLOPs (Floating-Point Operations)	Latency (s)	Time of Convergence (s)
MobileNet	13 million	16.89 million	0.01689	422,074
VGG16	138 million	16.81 million	0.01681	364,129
ResNet50	>23 million	49.90 million	0.04990	151.33

**Table 7:** Connectivity time results for Raspberry Pi, Alpha 1, and BlueSPP Android Apps.

Device/Application	Raspberry Pi/PyBluez Library	Alpha 1 Android App	BlueSPP Android App
**Connectivity time (seconds) to Alpha 1P Robot**	3.23	28.18	3.8
**Bluetooth version**	Bluetooth 2.0	Bluetooth 5.0	Bluetooth 5.0
**Bluetooth version of Alpha 1P robot**	Bluetooth 3.0/4.0 BLE + EDR		

Abbreviations: BLE = Bluetooth low ernergy; EDR = enhanced data rate.

**Table 8:** Latency results for execution time of Alpha 1P robot’s action.

Device/Application	Raspberry Pi/PyBluez library	Alpha 1 Android App	BlueSPP Android App
**Latency time (seconds) representing executing an action by Alpha 1P Robot**	3.66	31.22	5.0
**Bluetooth version**	Bluetooth 2.0	Bluetooth 5.0	Bluetooth 5.0
**Bluetooth version of Alpha 1P robot**	Bluetooth 3.0/4.0 BLE + EDR		

## Data Availability

Not applicable.

## Abbreviations

BLE	Bluetooth Low Energy
EDR	Enhanced Data Rate
LE	Low Energy
MAC	media access control
RFCOMM	Radio frequency communication
RS-232	Recommended Standard 232
TCP	Transmission Control Protocol
